# Construction and Validation of a Generational Identity Scale on Bangladeshi Older Adults

**DOI:** 10.3389/fpsyg.2021.703237

**Published:** 2021-08-05

**Authors:** Azharul Islam, Shamsul Haque

**Affiliations:** ^1^Department of Psychology, Jeffrey Cheah School of Medicine and Health Sciences, Monash University Malaysia, Bandar Sunway, Malaysia; ^2^Department of Educational and Counselling Psychology, University of Dhaka, Dhaka, Bangladesh

**Keywords:** generational identity, scale construction, factor structure, reliability, validity, Bangladesh

## Abstract

There is a lack of a psychometric tool for generational identity. We have conducted two studies involving Bangladeshi older adults who have witnessed the Bangladesh liberation war in 1971 to develop a new generational identity scale (GIS). The first study (*N* = 300) prepared an initial pool of 31 items and got them vetted by expert judges, which retained 21 items to form the provisional GIS (GIS-21). An exploratory factor analysis on GIS-21 excluded eight items and offered a two-factor solution: (i) identification with the generation and (ii) awareness of the generational importance. The second study (*N* = 176) ran a confirmatory factor analysis on the resulting GIS-13 and dropped another item to achieve a better model fit (*SRMR* =0.058, *GFI* = 0.986, *AGFI* = 0.980, and *NFI* = 0.980). The remaining 12-item GIS (GIS-12) showed excellent reliability (Mc Donald's omega = 0.898) and satisfactory temporal stability (*ICC* = 0.59, 95% *CI* = 0.27–0.77) over a 4-week interval. The scale's moderate correlation with another measure for generational identification demonstrates its convergent validity. Participants' transitional experience caused by the Bangladesh independence war in 1971 was also moderately correlated with the GIS-12 supporting further theoretical convergence of this scale. We recommend that researchers could use this scale on different populations and age groups upon appropriate validation.

## Introduction

*Generation* is a cohort of similarly aged people who experience shared historical events during the critical period of identity formation (Mannheim, 1952). However, *generational identity* (GI) refers to an individual's awareness of membership in a generational group and the group's significance to the individual (Joshi et al., 2010; Lyons et al., 2018). While research on generation lacks a robust theory and methodological rigour (cf., Campbell et al., 2015; Costanza and Finkelstein, 2015), GI has strong theoretical underpinnings, especially embedded in psychosocial development theory (Erikson, 1959), social identity theory (Tajfel, 1982), and self-categorisation theory (Turner et al., 1987). Nevertheless, there is a dearth of standardised tools to assess GI; researchers typically use either birth year, memory content or custom items, all of which lack internal as well as external validity. Hence, we aimed to develop a new GI scale (GIS) and validate it on Bangladeshi older adults, who participated in the Bengali nationalist movement in the late 1960s and the Bangladesh war of independence in 1971 during their formative age.

In recent decades, some scholars have studied generation by examining the distributions of autobiographical memories across the adult lifespan. They recorded autobiographical memories from participants, collected participants' ages at events, and then examined memory distributions across the lifespan and/or calendar year. Typically, they observed a historically graded pattern on memory recollection, leading to the assumption that generations exist and a generation can be defined by its shared memories (Schuman and Scott, 1989; Schuman and Rieger, 1992; Schuman et al., 1998; Conway and Haque, 1999; Holmes and Conway, 1999). For Eyerman and Turner (1998), generation survives by maintaining a collective memory of its origins, its historical struggles, and its prominent leaders and ideologists. Identification with a generation links to an individual's activity, assigned meaning, and common fate in later adulthood (Schuman and Scott, 1989; Holmes and Conway, 1999; Settersten, 1999; Weiss and Lang, 2012; Weiss, 2014).

The prime theoretical account is that generational membership influences the attitudes and behaviours of the individuals (Mannheim, 1952; Joshi et al., 2010; Lyons et al., 2018). It has been suggested that a shared historical event would affect the values, cognition, and behaviour of the entire generation (National Research Council, 2002; Costanza and Finkelstein, 2015). However, this effect would be different across individuals, simply because they are different in terms of how they have been raised, the socio-cultural environment they were exposed to, and the level of education they completed. There is evidence that historical and social events affect people differently, even though they grew up around the same time (Griffin, 2004; Alwin and McCammon, 2007; Lyons and Kuron, 2014), supporting the well-established theory of individual differences in experiencing and understanding historical events (Rudolph and Zacher, 2016).

There is a recent trend to study generation from an identity development perspective (Holmes and Conway, 1999; Finkelstein et al., 2001; Joshi et al., 2010; Urick, 2012; Lyons et al., 2018). GI has been conceptualised as an extension of social identity, primarily operationalised as an individual's awareness of being a social group member (Joshi et al., 2010; Lyons et al., 2018). Considering generation as an identity-based concept eliminates the complexity of studying generational effects on behaviour and cognition because it primarily relies on the individual's perception of her or his generation. Additionally, an identity-based approach allows us to study generational effects on individuals across cultures as well as the importance of historical events they have witnessed. Research has indicated that GI remains stable across the lifespan and manifests profoundly during the latter half of life (Conway, 1997; Finkelstein et al., 2001; Weiss and Lang, 2009, 2012; Weiss, 2014).

According to Erikson, adolescents of 12–18 years start exploring their independence and form a sense of self-identity, mainly through interacting with others in the society and culture they belong to (Erikson, 1959). This exploration allows an individual to develop other identities related to group membership in addition to individual identity. Schuman and Scott (1989) observed a distinct generational pattern in the responses of adult Americans when asked to report “the national or world events or changes over the past 50 years.” Different age groups recalled and rated different historical events as significant, primarily corresponding to the events that happened during participants' adolescence and early adulthood (Schuman and Scott, 1989). Historical events that happen during the critical development stages like this form significant collective memories (Dencker et al., 2008), which are then used to interpret personal memories (Conway, 1997; Schuman, 2004). GI is manifested in people's attitudes, beliefs, behaviour, and cognition that remains relatively stable throughout the lifespan (Strauss and Howe, 1991; Inglehart, 1997).

Settersten (1999) proposed three specific components of GI. First, the *relational* component, which states that a generation is always perceived in relation to other generations (“us” vs. “them”). Second, the *change* component captures the historical events and changes that shape a generation's identity. Third, the *convoy* aspect describes generations as “interactive systems of age peer relationships” that are present across the lifespan (Settersten, 1999). Likewise, Finkelstein et al. (2001) have found “emotional attachment and importance,” “value,” and “cognitive awareness” as three distinct but related factors in their age and generation identification scale. Taken together, various definitions indicate that at least four identifiable but related components constitute GI: (a) recognition of the existence of a generational group, (b) self-categorisation as a member of that group, (c) perception of importance or value in group membership, and (d) feelings or beliefs about the group and its distinctiveness from other generational groups.

### The Current Study

Several theoretical and critical reviews emphasised the need for collecting GI data directly from participants (Costanza et al., 2012; Costanza and Finkelstein, 2015; Zacher, 2015; Zabel et al., 2016; Lyons et al., 2018). For this, a validated psychometric tool is required, which could assess to what extent people identify themselves emotionally with their perceived generational group and how important they think that generation would be in their life (Finkelstein et al., 2001). The assessment of individual differences in GI is crucial as it allows the researchers to explore if people with stronger GI think and behave differently from those having weaker GI (Costanza et al., 2012; Van Rossem, 2018).

Although several GI assessment tools exist, they have several limitations (Finkelstein et al., 2001; Weiss and Lang, 2012; Weiss, 2014). For example, Finkelstein and colleagues developed a 24-item scale to assess “age and generation identification” together based on data collected from undergraduate psychology students (Finkelstein et al., 2001). However, combining age and generation in one scale is deemed problematic, as researchers often want to dissociate the effects of age and GI. Indeed, research has shown that with age, people develop a dual identity - an age-related identity and a generation-related identity (Weiss and Lang, 2009). Moreover, data from college students reduced the scale's external validity as evidence shows that, although people develop their GI within 16–25 years of age, its effect becomes apparent in the second half of life (Weiss and Lang, 2009, 2012; Weiss, 2014). Hence, we suggest that the construction of a GI scale should engage at least middle-aged participants to increase its external validity. There is another scale, which measures the GI of older adults, but this scale only focused on the cognitive aspect of identity (Tajfel, 1982). The internal consistency of this scale for older adults was also found insufficient (Cronbach's alpha = 0.60). We found three more scales that assessed group identification, collective self-esteem, and generational continuity. The group identification scales (Brown et al., 1986; Hinkle et al., 1989) do not necessarily measure the identity of a generational group; they typically assess in-group identification and inter-group difference for groups that can be formed in organisations, society, or community. The collective self-esteem scale (Luhtanen and Crocker, 1992) assesses an individual's social or collective identity, irrespective of the generation he or she belongs to. Finally, the need for generational continuity scale (Weiss, 2014) measures only the perceived awareness of the long-term effect of one's generation. The problem of this scale is that it measures only one dimension of generational identity (i.e., awareness), and it does not have adequate psychometric properties (only Cronbach's alpha was reported to be 0.86).

Due to the shortcomings of the currently available tools, we planned to construct a new standardised GI scale. For this, we conducted two studies involving Bangladeshi older adults who upraised politically during their formative years and participated in the Bengali nationalist movement in the late 1960s and the war of independence in 1971. This generation witnessed many socio-political and war events during a period when they attained their adult identity. With a shared vision, they fought for democracy, freedom, and social justice, and in that process, they emerged as a generation. Although they belong to one generational cohort, the extent to which they identify themselves with the cohort may vary. They may also rate the importance of being a member of that cohort differently. The current scale would be able to assess those individual differences. The first study prepared an initial pool of items and conducted an exploratory factor analysis to identify the scale's latent structure. The second study verified the factor structure that emerged from the first study using confirmatory factor analysis and established the scale's other psychometric properties.

## Study 1 Item Generation and Factor Structure of GIS Through Exploratory Factor Analysis

This study had three objectives. First, to generate items for the scale. Second, to get the items vetted by a panel of expert judges for their content validity. Third, to conduct an exploratory factor analysis to discover the latent structure of the scale.

## Method

### Participants

Data were collected as part of the large study exploring autobiographical memories of Bangladesh war veterans. Following the inclusion criteria (i.e., normal cognitive functioning and absence of significant physical and psychological illness), 332 Bangladeshi older adults were recruited from 10 Bangladesh districts. Thirty-two participants were excluded because their data were incomplete. The remaining data of 300 participants were analysed for this study. A sample of 300 has been recommended sufficient to explore the initial factor structure of a scale under construction (Henson and Roberts, 2006; Worthington and Whittaker, 2006; Comrey and Lee, 2013; Carpenter, 2018). Participants were recruited through poster invitations placed in areas where older people gather, such as community clubs and mosques. They were also approached through personal contacts and snowballing techniques. Out of 300 participants, 196 were men, ranging in age from 55 to 89 years (*M* = 66.35 years, *SD* = 5.91 years), and 104 women, ranging from 55 to 98 years (*M* = 66.88 years, *SD* = 6.69 years). All participants were married, and most of them were living with their spouses (70%). Almost all participants reported that they came from either middle or lower-middle-class families; more than half of them (59%) had no formal education.

### Procedure

#### Ethical Approval

The project obtained ethics clearance from the Monash University Human Research Ethics committee (Project ID: 11227). Before data collection, all participants signed the written consent form. We presented a written explanatory statement depicting the purpose and procedure of the study to the participants. Trained research assistants also verbally explained the participants' rights and the nature of tasks they were required to complete. Once they signed the written consent form, the data collection commenced, which took place from December 2019 to January 2020. Participation was voluntary.

#### Items Construction

After reviewing the literature, we found several generation- and identity-related measures: (1) Age and Generation Identification Scale (Finkelstein et al., 2001), (2) Group Identity Scales (Brown et al., 1986; Hinkle et al., 1989), (3) Generational Identification Measure (Weiss and Lang, 2009), (4) Need for Generational Continuity Scale (Weiss, 2014) and (5) Collective Self-esteem Scale (Luhtanen and Crocker, 1992). We selected items from these scales considering the face validity, use, and potential for covering the breadth of the GI construct. We edited the items to refer to a generation rather than a social group or self. Next, we eliminated items of similar meanings, resulting in a list of 31 mutually exclusive items (see Table 1). We preferred a five-point Likert-type response format over a dichotomous format (e.g., Yes/No) as “multiple-choice item formats are more reliable, offer more stable results, and produce better scales” (Comrey, 1988).

**Table 1:** List of Items for Generational Identity Scale.

**GIS-31**	GIS-21^*^	GIS-13^*^	GIS-12^*^
1. It is important for my generation to pass along the experiences we have undergone.	√	√	√
2. I think my generation is meaningful to many people.	√	√	√
3. I feel that my generation will be remembered for a long time.	√	√	√
4. My generation has made unique contributions to society.	√	√	√
5. My generation can pass along valuable ideas and experiences.	√	√	√
6. In general, belonging to my generation is an important part of my self-image.	√	−	−
7. I feel strong ties with the people of my generation.	√	√	−
8. I am glad to belong to my generation.	√	−	
9. I am annoyed to say I am a member of our generation.	√	−	−
10. I feel that my generation's contributions will continue to exist when we are gone.	√	−	−
11. My generation made and created things that have had an impact on other people.	√	−	−
12. I feel proud of my generation's achievements.	√	√	√
13. I feel good about my generation.	√	√	√
14. I identify myself with my generation.	√	√	√
15. I see me as belonging to my generation.	√	√	√
16. I try to hide belonging to my generation.	√	−	−
17. I do not fit in well/feel uneasy with other members of my generation	√	−	−
18. I am different from people of my generation.	√	−	−
19. I am a worthy member of the generation I belong to.	√	√	√
20. The generation I belong to is an important reflection of who I am.	√	√	√
21. I am a cooperative participant in the generation I belong to.	√	√	√
22. I consider my generation important.	−	−	−
23. I criticise our generation.	−	−	−
24. I do not consider my generation to be important.	−	−	−
25. Overall, my generational identity has very little to do with how I feel about myself.	−	−	−
26. I feel held back by my generation.	−	−	−
27. I feel I do not have much to offer to the generation I belong to.	−	−	−
28. I make excuses for belonging to my generation.	−	−	−
29. I often regret that I belong to my generation.	−	−	−
30. The generation I belong to is unimportant to my sense of what kind of a person I am.	−	−	−
31. I often feel I am a useless member of my generation.	−	−	−

* *Items retained after expert review (GIS-21), exploratory factor analysis (GIS-13), and confirmatory factor analysis (GIS-12)*.

#### Item Translation

Two independent bilingual translators, one of whom was a subject matter expert in psychology, translated all 31 items into Bangla. A language expert reviewed and synthesised the two versions of translations.

#### Item Selection: Expert Panel Review

We gave the whole list of 31 items to an expert panel to judge their relevance and representativeness of the GI construct. The panel was composed of three practising psychologists (a clinical psychologist, a counselling psychologist, an educational psychologist) and two academic psychologists. The panel members independently rated each of the items on a two-point scale for relevance (1 = Relevant, 0 = Not relevant), representativeness (1 = Representative, 0 = Not representative) and clarity (1 = Clear, 0 = Not clear). A score of 3 (satisfying all three criteria) was required for an item to be retained by all five raters. Ten items were dropped in this process, leaving only 21 items (GIS-21) for larger field-testing. It is worth noting that out of the 10 excluded items, nine were negative statements. The expert judges excluded those items mostly because they considered them to lack clarity for older people.

### Measures

#### Generational Identity Scale (GIS-21)

The GIS-21, the provisional GIS, was our target instrument for validation. Each item has a five-point Likert-type response option: 0 for “completely disagree,” 1 for “disagree,” 2 for “neutral,” 3 for “agree,” and 4 for “completely agree.” Scores of item 9, 16, 17, and 18 needed to be reversed as their framing was negative. We included these negatively worded items on the scale as we followed the traditional method of item construction to reduce systematic response bias. The total GI score was obtained by adding scores for all individual items; therefore, a higher score indicates a stronger GI.

#### Short Portable Mental Status Questionnaire

As we recruited older participants, it was possible that some of them would have some degree of cognitive impairment, which could potentially influence their responses to the GIS items. For example, a participant with significant memory loss might not be able to respond to the item “My generation has made unique contributions to society.” Therefore, we screened the participants before data collection. We used the SPMSQ (Pfeiffer, 1975), a 10-item tool to detect the degree of cognitive impairment among older participants. Each incorrect response to the SPMSQ item was scored 1, and the correct answer was scored 0. Therefore, the total score could range from 0 (no errors) to 10 (all errors), in which a higher score indicated a higher cognitive impairment. Pfeiffer (1975) suggested the following indexes for Caucasian participants with at least some high school education: intact cognition (0–2 errors), mild impairment (3–4 errors), moderate impairment (5–7 errors), and severe impairment (8–10 errors) in cognitive functioning. Considering the lower educational level of the present sample (e.g., many had no formal education), we considered up to five errors as mild cognitive impairment as suggested by Pfeiffer (Pfeiffer, 1975). The current sample comprised of participants who scored five or less on the SPMSQ.

### Analytic Strategies

Data were analysed using the program FACTOR (Ferrando and Lorenzo-Seva, 2017). Since the GIS items were scored on ordinal scales, and univariate distributions of those items were asymmetric (Table 2) we performed exploratory factor analysis (EFA) on polychoric correlations (Holgado–Tello et al., 2008; Rhemtulla et al., 2012). The internal structure of GIS-21 was examined using Robust Diagonally Weighted Least Squares (RDWLS) with the Direct Oblimin rotation method. This oblique rotation method was selected because the factors of GIS were expected to correlate (Costello and Osborne, 2005). The RDWLS extraction method has been recommended for data that do not meet the multivariate normality assumption (Fabrigar et al., 1999). Before EFA, necessary assumptions were checked. The first criterion was the adequate sample size. The widely used criterion for an adequate sample size for EFA is the subjects-to-variables ratio of 4:1 or 5:1 (Streiner, 1994; Floyd and Widaman, 1995). The number of participants in the current study was 14 times larger than the number of items (300 participants against 21 items), which is considered as “good” (Comrey and Lee, 2013). To determine the best factor structure for the GIS, we supplement the initial factor structure revealed from Eigenvalues above 1 with Monte Carlo's parallel analysis (Watkins, 2006) and Schwarz's Bayesian Information Criterion (BIC) Dimensionality test available in the FACTOR program. The FACTOR program provides several fit indices for EFA, including the comparative fit index (CFI), Tucker-Lewis non-normed fit index (NNFI), Weighted Root Mean Square Residual (WRMR), and the root mean square error of approximation (RMSEA). The CFI and NNFI indices are considered suitable at 0.90 and good at 0.95 (Bryant and Yarnold, 1995). The RMSEA and WRMR are considered suitable at values lower than 0.10 and good below 0.06 (Yu, 2002; Thompson, 2004). For item retention for each factor, we followed four best practises recommended by psychometricians: (i) no factors with fewer than three items, (ii) no items which cross-loaded greater than 0.3 across factors, (iii) no items with communality less than 0.3, and (iv) no items with a factor loading <0.4 (Clark and Watson, 1995; Worthington and Whittaker, 2006; Simms, 2008; Carpenter, 2018).

**Table 2:** Univariate descriptive statistics of the 21-Item GIS (*N* = 300).

**Item**	**Mean**	**95% Confidence interval**	**Variance**	**Skewness**	**Kurtosis** **(Zero centred)**
1	3.44	(3.33–3.55)	0.55	−1.79	4.61
2	3.29	(3.17–3.41)	0.65	−1.58	3.70
3	3.26	(3.13–3.39)	0.76	−1.19	1.08
4	3.38	(3.27–3.49)	0.58	−1.36	2.39
5	3.23	(3.13–3.33)	0.46	−0.77	1.06
6	3.12	(3.00–3.24)	0.66	−0.81	0.71
7	3.27	(3.14–3.40)	0.80	−1.53	2.45
8	3.26	(3.12–3.40)	0.92	−1.54	2.06
9	3.15	(2.99–3.30)	1.06	−1.38	1.46
10	2.98	(2.84–3.11)	0.80	−0.94	1.01
11	3.27	(3.16–3.38)	0.53	−1.14	2.14
12	3.42	(3.30–3.53)	0.61	−1.56	2.58
13	3.29	(3.17–3.41)	0.67	−1.66	3.80
14	3.12	(2.99–3.26)	0.87	−1.24	1.50
15	3.16	(3.05–3.27)	0.56	−0.90	1.07
16	3.22	(3.08–3.37)	0.93	−1.65	2.76
17	3.11	(2.97–3.25)	0.85	−1.32	1.90
18	2.57	(2.39–2.75)	1.50	−0.87	−0.28
19	3.23	(3.10–3.35)	0.75	−1.29	1.88
20	2.96	(2.82–3.09)	0.82	−0.80	0.38
21	3.03	(2.90–3.17)	0.85	−1.01	0.91

## Results and Discussion

### Descriptive Statistics and Item Analysis

Table 2 reports univariate descriptive statistics for the 21-items of the GIS (see Supplementary Material 1 for bar plots). Most of the items were skewed with high kurtosis. Although Mardia's (Mardia, 1970) test for skewness was not statistically significant (*p* = 1.00), there was evidence of high kurtosis, *p* < 0.001, necessitating the use of polychoric correlations instead of Pearson's correlations. Inter-item correlation coefficients were above 0.30 in most cases except for items 9, 16, 17, and 18. These four items showed extremely low correlations (Table 3). However, no item was discarded based on descriptive statistics or item analysis.

**Table 3:** Inter-item polychoric correlation coefficients for the 21-Item GIS (*N* = 300).

**Item**	**1**	**2**	**3**	**4**	**5**	**6**	**7**	**8**	**9**	**10**	**11**	**12**	**13**	**14**	**15**	**16**	**17**	**18**	**19**	**20**	**21**
1	1.00																				
2	0.62	1.00																			
3	0.64	0.69	1.00																		
4	0.60	0.59	0.73	1.00																	
5	0.62	0.56	0.69	0.73	1.00																
6	0.50	0.42	0.55	0.57	0.71	1.00															
7	0.52	0.40	0.42	0.50	0.49	0.55	1.00														
8	0.49	0.36	0.56	0.66	0.52	0.64	0.64	1.00													
9	0.19	0.15	0.32	0.34	0.17	0.32	0.25	0.54	1.00												
10	0.60	0.53	0.61	0.55	0.63	0.55	0.43	0.55	0.17	1.00											
11	0.64	0.57	0.57	0.55	0.68	0.53	0.51	0.55	0.09	0.70	1.00										
12	0.56	0.43	0.56	0.63	0.54	0.48	0.58	0.73	0.36	0.60	0.73	1.00									
13	0.58	0.47	0.60	0.60	0.65	0.59	0.59	0.70	0.32	0.56	0.68	0.81	1.00								
14	0.51	0.43	0.51	0.62	0.55	0.53	0.61	0.57	0.20	0.48	0.52	0.66	0.70	1.00							
15	0.54	0.45	0.45	0.58	0.58	0.55	0.53	0.56	0.20	0.51	0.56	0.62	0.67	0.71	1.00						
16	0.16	0.04	0.21	0.20	0.14	0.25	0.21	0.38	0.71	−0.01	−0.03	0.31	0.31	0.16	0.13	1.00					
17	0.30	0.17	0.32	0.34	0.28	0.30	0.32	0.32	0.56	0.19	0.20	0.35	0.30	0.29	0.29	0.66	1.00				
18	0.22	0.27	0.27	0.24	0.32	0.14	0.12	0.09	0.33	0.20	0.31	0.24	0.20	0.16	0.19	0.32	0.49	1.00			
19	0.44	0.35	0.49	0.54	0.51	0.55	0.55	0.64	0.32	0.54	0.56	0.64	0.66	0.58	0.62	0.22	0.30	0.19	1.00		
20	0.55	0.49	0.53	0.60	0.67	0.61	0.59	0.60	0.18	0.66	0.67	0.63	0.66	0.67	0.68	0.09	0.32	0.21	0.79	1.00	
21	0.49	0.39	0.45	0.47	0.59	0.60	0.56	0.59	0.22	0.59	0.56	0.62	0.65	0.62	0.73	0.15	0.25	0.13	0.76	0.79	1.00

### Exploratory Factor Analysis

The initial inspection of the *R*-matrix (Table 3) indicated that a large number of the coefficients (78.10%) were above 0.30. The Kaiser-Mayer-Olkin (KMO) index was 0.90, exceeding the recommended value of 0.60 (Kaiser, 1970), and Bartlett's Test of Sphericity (Bartlett, 1954) reached statistical significance (χ^2^ = 3,352.2, *p* < 0.001), indicating that our data were suitable for factor analysis. The results of the initial analysis revealed three factors with Eigenvalues over 1, explaining 51.27, 10.89, and 6.64% of the variance, respectively. The BIC dimensionality test also indicated a three-factor solution. However, a parallel analysis with 21 items, 300 participants with 500 replications indicated a two-factor solution for the scale (Watkins, 2006). We tested both the three and two-factor solutions. First, the EFA with three-factor revealed a lack of fit in the model, in which one item (number 18) showed poor loading (<0.40), low communality, cross-loaded across factors. Three other items (number 6, 10, 11) also cross-loaded across factors. Therefore, these four items were deemed problematic to the factor structure of the GIS. After discarding these items, we ran the EFA with the remaining 17 items maintaining the three-factor solution. This model also had a poor fit with one item (number 8) that cross-loaded across factors. A revised EFA with 16 items after discarding the cross-loaded item resulted in an ambiguous three-factor solution in which only three items loaded on a third factor, and the remaining 13 items formed the two factors (one factor contained five items, another included eight items). The goodness of fit of this model was acceptable, but the third factor was poorly associated with the first (*r* = 0.298) and second (*r* = 0.280) factors. To detect the potential misfit, we inspected the three items forming the third factor. We found that item number 9 (I am annoyed to say I am a member of our generation), 16 (I try to hide belonging to my generation), and 17 (I do not fit in well/feel uneasy with other members of my generation) were negatively framed and perhaps appeared vague to our participants. Literature showed that the inclusion of negatively worded items in a scale might lead to spurious factors containing only these items (Zhang et al., 2016). This is due to a method effect and how participants respond to negative items rather than representing true factors. Therefore, we dropped this low correlated factor in order to improve clarity and brevity of the scale and considering the elderly participants, the majority of whom had no formal education (Benson et al., 2020).

After discarding these three items, we ran a further EFA holding two-factor (as suggested by parallel analysis) and found a parsimonious model with 13 items. This model gave a clean two-factor solution complying with all the item-retention criteria. Eight items (number 7, 12, 13, 14, 15, 19, 20, and 21) loaded on the first factor, and five items (number 1, 2, 3, 4, and 5) loaded on the second factor (Table 4). These two factors jointly explained 71.27% of the variance. Inspection of the items indicated that factor one could be termed as “identification with the generation,” and factor two as “awareness of the generational importance.” The correlation between the two factors was 0.69. The internal consistency reliability of the 13-item GIS (McDonald's omega = 0.922) and its factors (Factor 1 omega = 0.907 and Factor 2 omega = 0.838) were excellent.

**Table 4:** Standardised factor loadings from study 1 (EFA, *N* = 300) and study 2 (CFA, *N* = 176).

**Items**	**Study 1 (EFA) loadings**		**Study 2 (CFA) loadings**	
	**Identification**	**Awareness**	**Identification**	**Awareness**
1) It is important for my generation to pass along the experiences we have undergone.	0.17	**0.64**		0.74
2) I think my generation is meaningful to many people.	−0.06	**0.80**		0.66
3) I feel that my generation will be remembered for a long time.	−0.07	**0.93**		0.77
4) My generation has made unique contributions to society.	0.17	**0.73**		0.81
5) My generation can pass along valuable ideas and experiences.	0.25	**0.63**		0.57
7) I feel strong ties with the people of my generation.	**0.56**	0.16	–	
12) I feel proud of my generation's achievements.	**0.62**	0.24	0.73	
13) I feel good about my generation.	**0.63**	0.26	0.61	
14) I identify myself with my generation.	**0.65**	0.18	0.68	
15) I see me as belonging to my generation.	**0.74**	0.09	0.76	
19) I am a worthy member of the generation I belong to.	**0.90**	−0.07	0.78	
20) The generation I belong to is an important reflection of who I am.	**0.82**	0.08	0.82	
21) I am a cooperative participant in the generation I belong to.	**0.99**	−0.14	0.68	

*Method of factor extraction: Robust Diagonally Weighted Least Squares (RDWLS), Method of rotation: Direct Oblimin. Note. Loading >0.4 are in bold font*.

## Study 2 Confirmation of Factor Structure and Psychometric Properties of GIS-12

This study had three objectives. First, to confirm the factor structure of GIS-13, which emerged from the EFA in the first study, through confirmatory factor analysis (CFA). Second, to examine the internal and external validity of the scale (Wasserman and Bracken, 2003). We explored the internal validity of GIS-13 using its content validity and the scale's internal structure. To check the scale's convergent validity, we calculated the bivariate correlation between the scores of GIS and generational identification measure (GIM) and Transitional Impact Scale (TIS). Third, to establish reliability by assessing the internal consistency and test-retest reliability. Test-retest reliability was assessed by correlating scores of GIS obtained from a smaller group of participants twice in 4 weeks. Intra-class Correlation Coefficients (ICC) with 95% confidence intervals were calculated to check the absolute agreement between time 1 and time 2.

## Method

### Participants

Following the same inclusion criteria and recruitment procedure used in Study 1, a second group of 200 Bangladeshi older adults was recruited for Study 2. Data of 24 participants were incomplete, so they were excluded from the study, resulting in a sample of 176; 20.5% women, ranging in age from 60 to 72 years (*M* = 63.92 years, *SD* = 3.92 years), and 79.5% men, ranging in age from 55 to 84 years (*M* = 67.84 years, *SD* = 5.53 years). We followed the widely recommended criterion for sample size calculation for CFA, which is participants to the number of parameters ratio with a minimum of 5:1 to a maximum of 10:1 (Bentler and Chou, 1987; Worthington and Whittaker, 2006). Our sample size exceeded the maximum requirement of 130, as we had 13 items on the scale. All participants were married, and the majority of them were living with their spouses (86.4%). Almost all participants came from either middle or lower-middle-class families; 75% completed at least primary education, while 23% had no formal education. Forty-nine participants were retested 4 weeks apart to examine the scale's temporal stability.

### Measures

#### Generational Identity Scale-13 (GIS-13)

The GIS-13 was derived from the EFA conducted in Study 1 with two factors. The scale and its two factors all had excellent internal consistency.

#### Generational Identification Measure

The GIM is a 4-item scale to measure an individual's sense of belonging and being part of one's generation (Weiss and Lang, 2009). Items are “I identify with people of my generation,” “I feel strong ties with people of my generation,” “I am different from people of my generation,” and “I feel a sense of belonging to people of my generation.” The GIM has been used to assess the convergent validity of the scale under construction.

#### Transition Impact Scale

The current sample experienced the Bangladesh independence war in 1971 during their formative years, a period antecedent of forming generational identity (Holmes and Conway, 1999). It was hypothesised that participants who experienced a higher transitional impact due to the independence war would form a stronger generational identity at present (Lyons et al., 2018). To assess the changes the war of independence brought to the participants' lives, we used the 12-item Transitional Impact Scale (TIS, Svob et al., 2014). TIS consists of two factors: material change and psychological change, each comprising six items. Each item was scored on a five-point Likert scale ranging from 1 (Completely Disagree) to 5 (Completely agree). Responses of six material change items and six psychological change items were averaged to get a material change score and a psychological change score, respectively. TIS was translated into Bangla following standard procedure (e.g., forward-backward translation and judged by an expert panel). We replaced the phrase “This event” with “War of independence” for each item, for example, “The war of independence has changed where I live” (material change) and “The war of independence has changed the way I think about things” (psychological change). The goodness of fit of the two-factor solution for the Bangla TIS-12 was checked using confirmatory factor analysis with maximum likelihood estimation (χ^2^*/df* = 142/53 = 2.67, *CFI* = 0.92, *TLI* = 0.89, *RMSEA* = 0.09, and *SRMR* = 0.63). The Bangla TIS also demonstrates excellent internal consistency (Cronbach's alpha for full scale = 0.89, material change = 0.85, and psychological change = 0.86).

### Procedure

Ethics clearance for this study was obtained together with Study 1. Trained research assistants briefed the research procedure to the participants. Once agreed, participants signed the consent form, upon which the data collection commenced. The administration of the three scales was randomised. The data collection took place at the participants' preferred locations, and their participation was voluntary.

## Results and Discussion

### Development of the Final GIS

**Figure 1:**
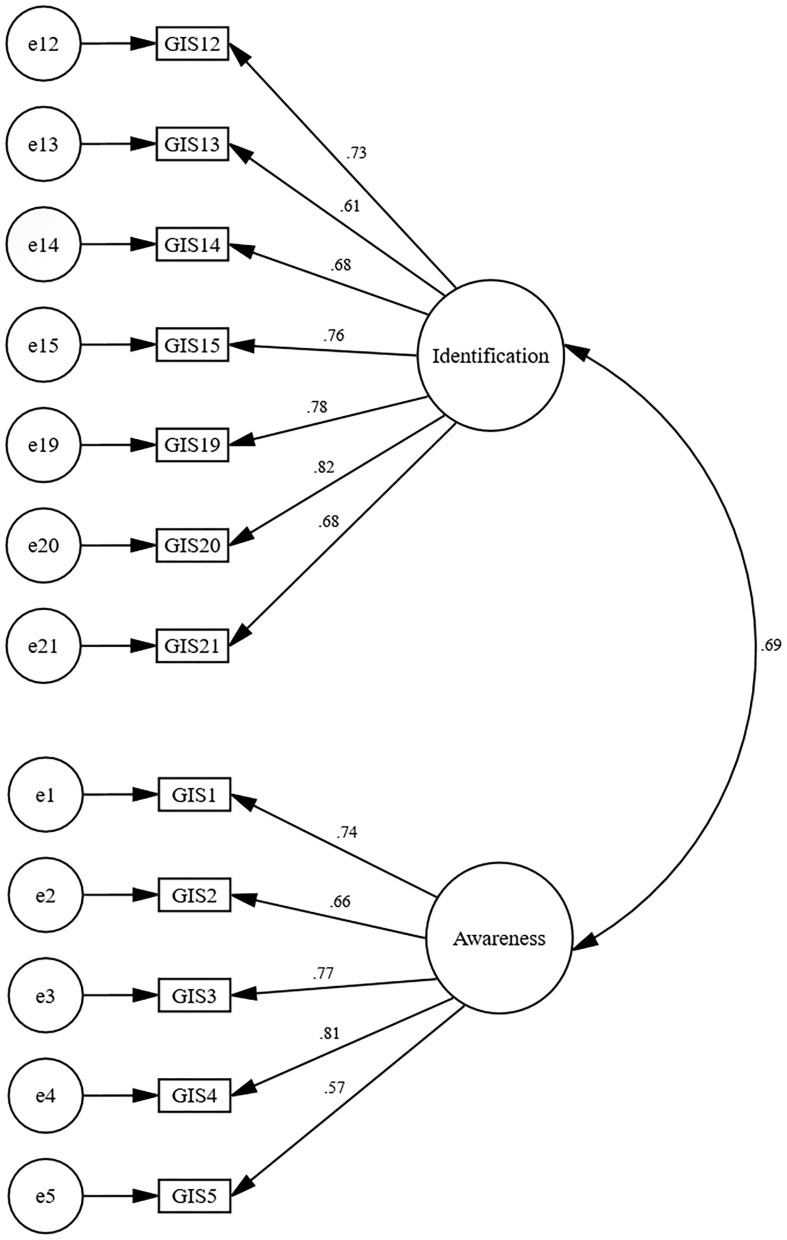
Factor structure of the two-factor solution for the Generational Identity Scale (Standardised parameter) (*N* = 176).

The goodness of fit of the two-factor solution for the GIS-13 was cross-checked using a CFA with Scale Free Least Squares (SFLS) estimation method. SFLS was chosen due to the ordinal response options being used for the scale items. To assess the model fit, we have reported commonly used fit indices: (i) standardised root-mean-square residual (SRMR), (ii) the Goodness of Fit Index (GFI), (iii) Adjusted Goodness of Fit Index (AGFI), (iv) Normed Fit Index (NFI). The SFLS does not produce a Chi-square test result or RMSEA value. SRMR value of <0.06, and GFI/AGFI and NFI values exceeding 0.95 have been suggested as indicative of a best-fitted model (Hu and Bentler, 1999; Brown and Moore, 2012). The fit indices presented in Table 5 indicate that the two-factor solution for GIS-13 seems acceptable, but the value of SRMR was slightly higher than the reference point. We inspected item loading to improve the model fit and found that one item (number 7) loaded poorly. When we ran the CFA after discarding item 7, the revised model was an excellent fit of the data (Table 5). Internal consistency reliability also improved after removing item 7. Therefore, we decided to discard item 7 from the final version of the scale, leading to a 12-item GIS (GIS-12), in which seven items loaded on Factor 1 and five items loaded on Factor 2 (Figure 1). See Supplementary Material 2 for the Bangla and English versions of the final scale with the scoring procedure.

**Table 5:** Summary of fit indices for the GIS confirmatory factor analysis models (*N* = 176).

**Model**	**SRMR**	**GFI**	**AGFI**	**NFI**	**Internal consistency** **(McDonald's omega)**
Two-factor (GIS-13 items)	0.064	0.982	0.975	0.974	0.895
Two-factor (GIS-12 items)	0.058	0.986	0.980	0.980	0.898

*GIS, Generational Identity Scale; SRMR, Standardised root mean square residual; GFI, Goodness of Fit Index; AGFI, Adjusted Goodness of Fit Index; NFI, Normed Fit Index*.

### Reliability of GIS-12

The reliability of GIS-12 was calculated through internal consistency and test-retest reliability.

#### Internal Consistency

Internal consistency specifies the degree to which the items of GIS-12 were inter-correlated (Clark and Watson, 1995). We reported McDonald's omega, an equivalent of Cronbach's alpha for ordinal data, as a measure of internal consistency (Zumbo et al., 2007). The omega values demonstrate adequate internal consistency for the scale as well as its two factors (Table 6).

**Table 6:** McDonald's omega and test-retest reliability of the GIS-12 and its factors.

**GIS**	**McDonald's omega**	**Test-retest reliability**	
		**Bivariate correlations**	**Intra-class correlation**
GIS-12	0.90	0.56^**^	0.59
Factor 1 (Identification)	0.89	0.45^**^	0.46
Factor 2 (Awareness)	0.84	0.32^**^	0.49

** *p < 0.01, 2-tailed*.

#### Test-Retest Reliability

**Figure 2:**
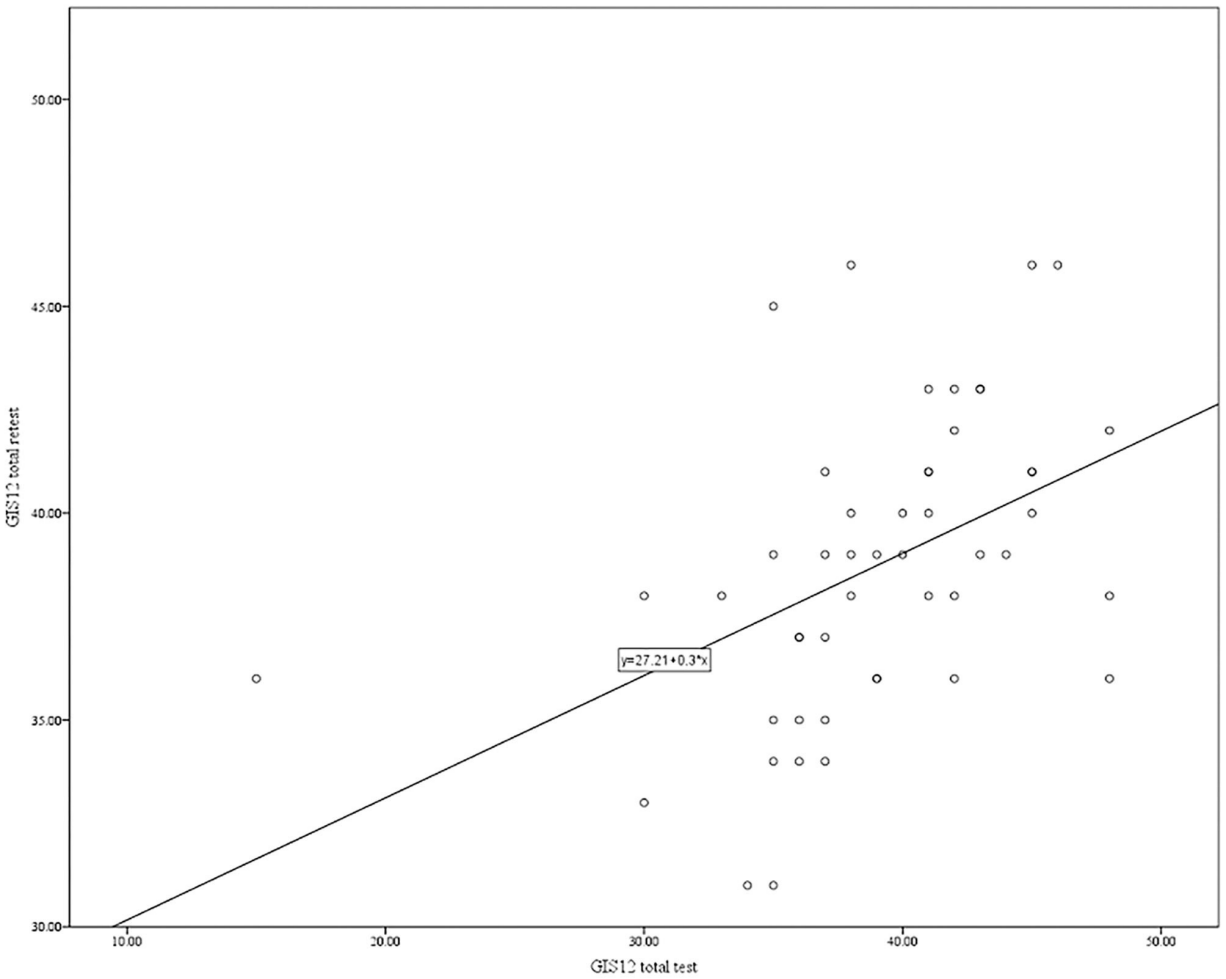
The relationship between the GIS-12 scores obtained at Time 1 and Time 2.

Test-retest reliability (temporal stability) of the GIS-12 was assessed through correlational analysis between GIS-12 scores obtained from 49 participants twice 4 weeks apart. The correlation coefficient was 0.56 (*p* < 0.001), indicating the scale's moderate temporal stability. However, since the bivariate correlation coefficient does not take into account the systematic differences, researchers have recommended the Intra-class Correlation Coefficient (ICC) as a standard parameter for absolute agreement (Terwee et al., 2007). For an 80% power with alpha fixed at 0.05, a minimum sample size of 22 is recommended to detect the ICC value of 0.50 (Bujang and Baharum, 2017). In case of possible dropout at the retest phase, a further twenty per cent of the minimum sample is recommended, giving a target of 27 participants. Therefore, the recruitment of 49 participants in this study was adequate to establish the scale's temporal stability. The absolute agreement between scores at time 1 and time 2, ICC with 95% confidence intervals, was calculated based on a mean-score (*k* = 2) of the GIS-12, 2-way random-effects model. The ICC was 0.59, 95% CI (0.27–0.77), representing a moderate agreement (McGraw and Wong, 1996; Koo and Li, 2016). Figure 2 shows the scatterplot for the relationship between the GIS-12 scores obtained at time 1 and time 2.

### The Validity of GIS-12

Both internal and external validity of GIS-12 were checked (Wasserman and Bracken, 2003). We present internal validity by means of the scale's internal structure. As for external validity, we checked whether the scale correlates with GIM and TIS positively (i.e., convergent validity). Convergent validity was further assessed by examining each factor's Average Variance Extracted (AVE) (a summary indicator of convergence). An AVE value of 0.5 or more is considered acceptable (Hair et al., 2013).

We found scores obtained from GIS-12 and GIM (Weiss and Lang, 2009) were positively correlated, *r* = 0.69, *p* < 0.001, demonstrating the scale's satisfactory convergent validity. Scores on both factors of the scale also correlated positively with GIM scores (*r* = 0.72 for Factor 1 and *r* = 0.50 for Factor 2, in both cases *p* < 0.001). Item 14 and 15 in the GIS-12 were almost identical with the two items in the GIM scale. We inspected the correlation between the two scales after removing these two items from the GIS. The results showed that the 10-item GIS was positively and moderately correlated with GIM (*r* = 0.62, *p* < 0.001). Spearman (1904) recommended additional calculations to correct the correlation because the scores would typically contain measurement errors. However, we found mixed evidence for when one should perform Spearman's correction for attenuation to establish the true convergent validity of a scale. In many instances, Spearman's corrections overestimated correlations (exceeded the value 1.0) in two cases: first, when the sample size was relatively smaller, and second when the reliability coefficient of one or both the measures was low (Zimmerman and Williams, 1997; Charles, 2005; Spiegelman et al., 2005). In the current study, the reliability coefficient for GIS-12 is 0.90, and the reliability coefficient for GIM is 0.46. When we put these two reliability values in the denominator of Spearman's correction formula, the corrected correlation coefficient was almost 1.0. This overestimation is due to the low reliability of the GIM, which is also consistent with the literature. Therefore, applying the correction is not appropriate here.

As expected, the GIS also positively and moderately correlated with the degree of transitional impact caused by the independence war (*r* = 0.66, *p* < 0.001) and its factors (*r* = 0.53 for Factor 1 and *r* = 0.67 for Factor 2, in both cases, *p* < 0.001), indicating participants experiencing stronger transitional impact tend to form higher generational identity. Lastly, AVE for Factor 1 was 0.53 and for Factor 2 was 0.51, both exceeding the acceptable threshold (Hair et al., 2013), further demonstrating the scale's convergent validity.

## General Discussion

While developing GIS-12, we followed three standard recommendations that a scale should have substantive validity, structural validity, and external validity (Loevinger, 1957; Clark and Watson, 1995; Simms, 2008). The GIS-12 has substantive validity because it is rooted in the established theories of psychology, sociology, and organisational behaviour (Mannheim, 1952; Schuman and Scott, 1989; Lyons et al., 2018). The scale has also been developed following standard guidelines for scale construction. The structural and external validity of the scale have been established through statistical procedures.

We started with 31 mutually exclusive items (Study 1) gathered from the currently available scales measuring similar constructs (Brown et al., 1986; Hinkle et al., 1989; Luhtanen and Crocker, 1992; Finkelstein et al., 2001; Weiss and Lang, 2009; Weiss, 2014). After excluding ten items by the expert panel, we formed the provisional GIS with 21 items, which was administered on a large sample of senior Bangladeshi nationals. Item and factor analysis excluded another eight items, including all four negatively worded items, leading to a 13-item GIS. The exclusion of negatively worded items was quite in line with the results in the previous research that negative items reflect personality characteristics more than they measure the intended constructs (Johnson et al., 2004; DiStefano and Motl, 2006; Gu et al., 2015; Zhang et al., 2016). For example, while responding to item number 17 (I do not fit in well/feel uneasy with other members of my generation), a participant may exhibit his/her personality, such as introversion, rather than reflect authentic feeling about the generation. An EFA offered a two-factor solution for GIS-13. Eight items loaded to the first factor and five items loaded to the second factor.

A CFA on a separate sample (Study 2) confirmed a two-factor solution with an excellent model fit. However, one item (item 7) had to be discarded from GIS-13. Therefore, we confirmed a 12-item GIS with two distinct factors. Inspection of these items suggested that these factors are (i) identification with the generation and (ii) awareness of the generational importance. For example, items in Factor 1 were expressive of how participants feel and identify themselves with their generation. Items in Factor 2 were expressing the perceived significance of the generation.

For the current sample, generational identity is manifested in their perceived degrees of identification with their generational group and awareness about the significance of the generation. This evidence is consistent with the contemporary conceptualisation of GI, which argues for multiple components (Finkelstein et al., 2001; Lyons et al., 2018). Although Finkelstein and colleagues argued for a third component called “value,” we notice that the essence of the value component is embedded in the identification with generation factor, as the item “I feel good about my generation” representing value in Finkelstein et al. (2001), constitutes the identity factor in the current scale.

Several other scholars have suggested four components of GI: (a) recognition of the existence of a generational group; (b) self-categorisation as a member of that generational group; (c) perception of importance or value in group membership; and (d) feelings or beliefs about the group and its distinctiveness from other generational groups (Settersten, 1999; Finkelstein et al., 2001; Lyons et al., 2018). We found some overlap between the theorised components themselves. For example, the terms “self-categorisation as a member of a generation” and “recognition of generation” are somewhat similar, quite tricky to segregate, especially for the current participants. Similarly, the concepts of “the importance of generation” and “beliefs about the generation” carry almost similar meaning. The two components that have emerged from the current study are, therefore, justifiable.

We also found that the GIS-12 has satisfactory convergent validity as it is moderately correlated with the GIM developed by Weiss and Lang (2009). This moderate correlation indicates good convergent validity because neither low correlation nor high correlation is desired in the examination of convergent validity. If the correlation is too high, the newly developed scale is simply a duplicate instrument. If the correlation is too low, the scales are likely to measure entirely different constructs. The identification with the generation and the awareness of generational importance dimensions of the scale also have satisfactory convergent validity as they both moderately correlated with the GIM. Whereas, the GIM taps only the cognitive aspect of identity, GIS-12 measures the degree of identification with the generation and awareness of the generational importance. The degree of identification is particularly critical because this factor demonstrates individual differences. For example, an individual may be aware of the generational importance but may not feel identified with the group as much as other members do. Therefore, researchers may draw wrong inferences without assessing the degree of identification, such as generation Xers are “cynical and detached” (Crowley, 2003).

In a separate analysis, the GIS-12 moderately correlated with the transition impact scale. The scores on the two dimensions of the transition impact scale also positively correlated with the GIS-12, showing their separate convergent validity. The reason why our elderly participants demonstrated varied transitional impact and its association with GI could be attributed to the theoretical propositions of Lyons et al. (2018). According to Proposition 13, “shared memories of historical events occurring in a cohort's adolescence and early adulthood are antecedent to GI formation” (p. 10). The participants in the current study witnessed major political turmoil during their adolescence and early adulthood when the Bengali people organised nationwide protests against military dictators in the late 1960s and participated in the war of independence in 1971. Therefore, participants should have formed their GI during that period.

However, according to Proposition 3, “GI will be strongest in individuals who have had frequent exposure to generational discourse and found it to plausibly explain social phenomena” (Lyons et al., 2018). Although all participants survived the independence war, the material and psychological impacts were not equal. Some of the participants directly fought for the war, some had to take refugees in the neighbouring country, yet some were relatively less affected, as revealed in TIS scores. Therefore, we anticipated that participants who experienced a stronger transitional impact would form a stronger sense of GI.

The internal consistency of the scale and its two factors, as measured by McDonald's omega, were excellent. The GIS-12 also holds temporal stability over a 4-week interval–a decisive feature of the construct. We recommend that researchers translate the GIS-12 in various languages and use it for cross-cultural research upon appropriate validation. The scale has the potential for use in psychological, sociological, political, and organisational behaviour research. For example, scholars in cognitive psychology can explore if individuals with varied levels of GI show distinctive patterns of recalling memories of public and private life experiences. Scholars in consumer and organisational behaviour can use this scale to investigate if identification with a particular generation affects an individual's intention to buy a particular product. They can also explore whether employees with a stronger identification with a generation are more likely to hold increased organisational commitment than those with lesser identification with the generation.

### Future Directions

We recommend some works for future researchers. First, the GIS can be rolled out to various age groups to see if its psychometric properties remain the same across the age groups. Second, factorial invariance can be established for men and women as well as other age groups. Third, other identity measures with sound psychometric properties could be used to verify the scale's validity as GIM used in the current study had poor reliability. Fourth, future research can examine the discriminant validity of the GIS-12.

## Conclusion

As there is a lack of a psychometric tool for generational identity, we have developed a 12-item generational identity scale (GIS-12) with adequate psychometric properties. Two studies have established the scale's content validity, convergent validity, internal consistency, and test-retest reliability. The robust convergent validity (*r* = 0.69) is the scale's unique strength, which is not too high and not too low. The scale has two factors: (i) identification with the generation and (ii) awareness of the generational importance. We recommend GIS-12 as a standard tool that can replace the current methods of assessing GI, for example, the birth year category, the content of shared memories, or custom items. The items of GIS-12 are comprehensive, a potential strength for rolling this scale out to other age groups. Besides this, future research could utilise GIS-12 to assess the generational identity of different inter-and intra-generational samples to test various hypotheses in different fields of psychology and organisational behaviour. Upon appropriate validation, researchers can also use it in cross-cultural research on generational identity.

## Data Availability Statement

The raw data supporting the conclusions of this article will be made available by the authors, without undue reservation.

## Ethics Statement

The studies involving human participants were reviewed and approved by Monash University Human Research Ethics committee (Project ID: 11227). The patients/participants provided their written informed consent to participate in this study.

## Author Contributions

AI and SH: conceptualization and project administration. AI: data curation, formal analysis, and writing—initial draft. SH: funding acquisition, supervision, and writing—review and editing. All authors contributed to the article and approved the submitted version.

## Conflict of Interest

The authors declare that the research was conducted in the absence of any commercial or financial relationships that could be construed as a potential conflict of interest.

## Publisher's Note

All claims expressed in this article are solely those of the authors and do not necessarily represent those of their affiliated organizations, or those of the publisher, the editors and the reviewers. Any product that may be evaluated in this article, or claim that may be made by its manufacturer, is not guaranteed or endorsed by the publisher.

## Supplementary Material

Supplementary Material 1 Further details of the analyses performed.

Supplementary Material 2 Bangla and English version of the Generational Identity Scale (GIS-12).
