# Supplementary material for: Construction and Validation of a Generational Identity Scale on Bangladeshi Older Adults
Source: Front Psychol. 2021 Aug 5;12:703237. doi: 10.3389/fpsyg.2021.703237 (PMC8376147; doi:10.3389/fpsyg.2021.703237)
Supplement: Supplementary Material 1 — Further details of the analyses performed. [file Data_Sheet_1.docx]

# Supplemental Materials 1

##
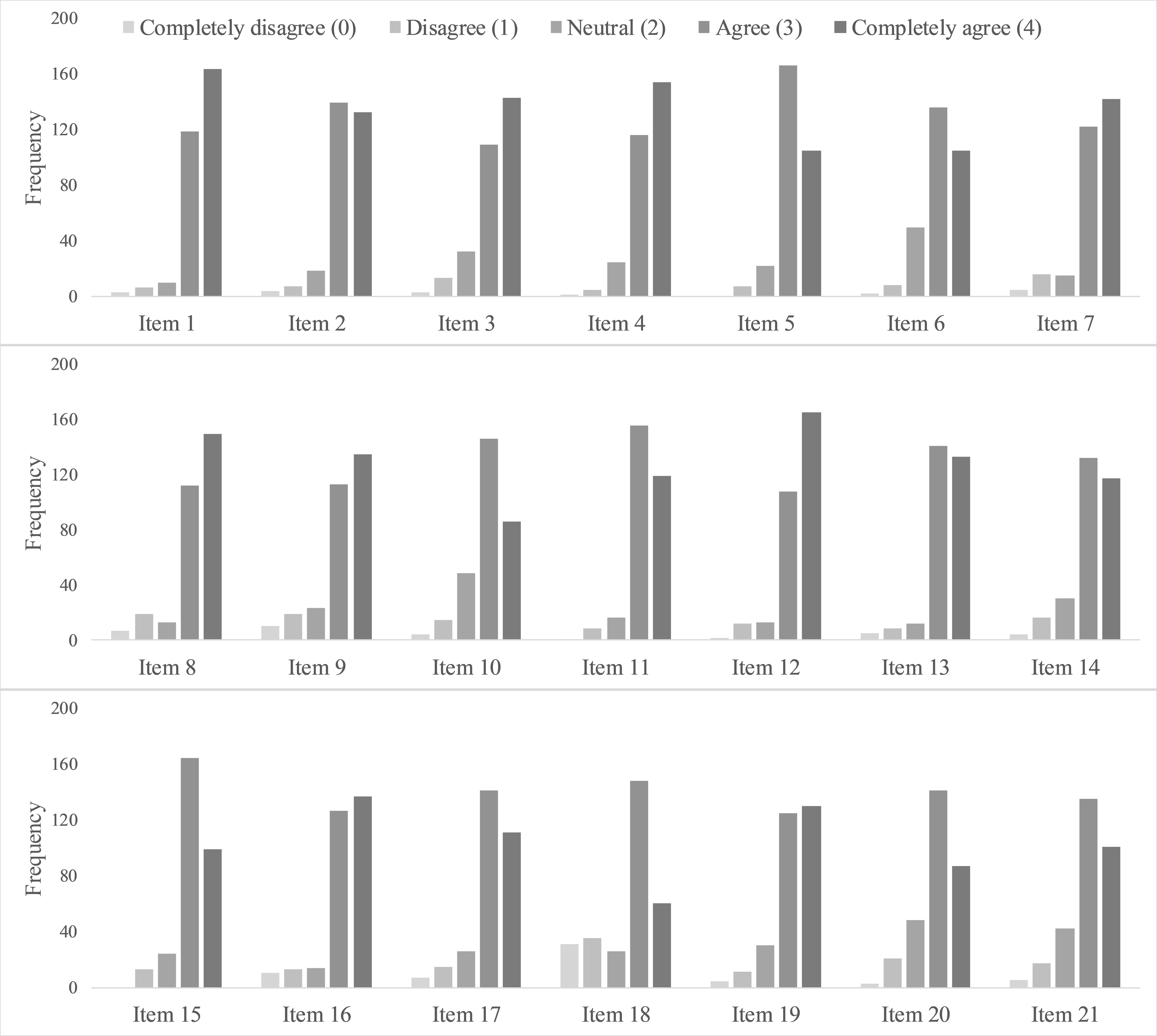
Bar plots GIS 21 items (Study 1)

**S1 Fig1. Bar Plots for the 21 Items of the GIS (Study 1, *N*= 300)**

## Bar plots GIS 12 items (Study 2)

**
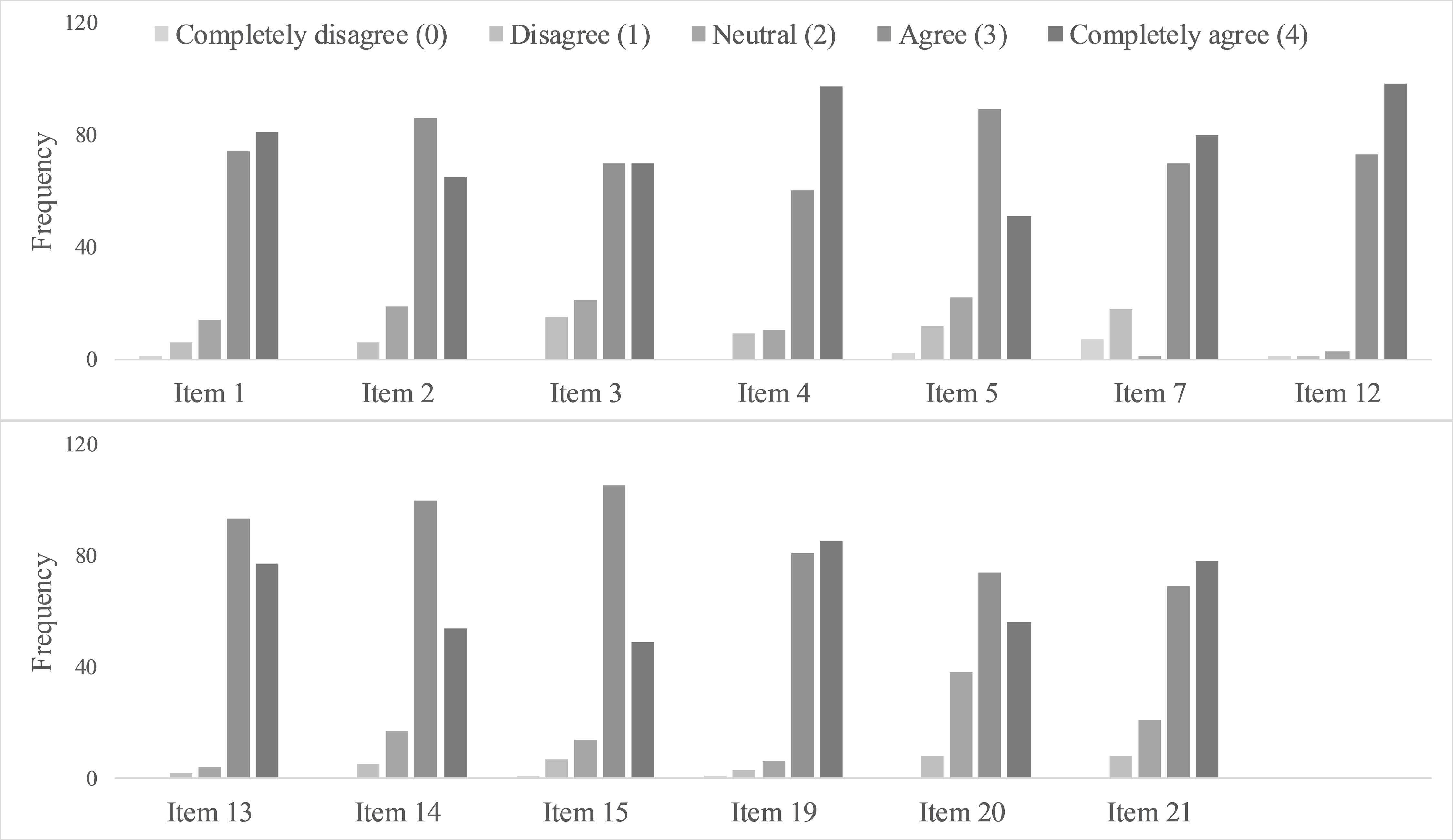
S1 Fig2. Bar Plots for the 13 Items of the GIS (Study 2, *N*=176)**

## Exploratory Factor Analysis (Study 1)

**S1 Table 1. Development of GIS through Exploratory Factor Analysis (Fit indices, and issues with GIS versions) (*N*=300)**

| **Versions** | **# of factor** | **RMSEA** | **WRMR** | **CFI** | **NNFI** |  | **Issues with Individual Items** |
| --- | --- | --- | --- | --- | --- | --- | --- |
| 21 items | 3 | .036 | .045 | .997 | .995 |  | Low loading item (18)  Low communality item (18)  Cross-loading item (6, 10, 11, 18) |
| 17 items | 3 | .042 | .041 | .996 | .994 |  | Cross-loading item (8) |
| 16 items | 3 | .039 | .038 | .997 | .995 |  | Negatively framed items (9, 16, 17) had poor associations with other factors |
| 13 items | 2 | .055 | .043 | .995 | .993 |  | All fine  Factor 1: 7, 12, 13, 14, 15, 19, 20, 21  Factor 2: 1, 2, 3, 4, 5 |

*Note*. CFI=Comparative fit index, NNFI=non-normed fit index, WRMR=Weighted Root Mean Square Residual (WRMR) , RMSEA= Root mean square error of approximation; Method of factor extraction: Robust Diagonally Weighted Least Squares (RDWLS) with polychoric correlations; Method of rotation: Robust Direct Oblimin
