# Supplementary material for: Construction and Validation of a Generational Identity Scale on Bangladeshi Older Adults
Source: Front Psychol. 2021 Aug 5;12:703237. doi: 10.3389/fpsyg.2021.703237 (PMC8376147; doi:10.3389/fpsyg.2021.703237)
Supplement: Supplementary Material 2 — Bangla and English version of the Generational Identity Scale (GIS-12). [file Data_Sheet_2.pdf]

Supplemental Materials 2

Generational Identity Scale (Bangla version)

নির্দেশনাঃ

নিচে একজন ব্যক্তির প্রজন্ম পরিচয় সম্পর্কিত কিছু বাক্য রয়েছে। ‘প্রজন্ম’ বলতে একটি নির্দিষ্ট সময়ে জন্ম ও বেড়ে ওঠা কোনো দলকে বোঝানো হচ্ছে, যারা সাধারণত একই ঐতিহাসিক, সামাজিক, রাজনৈতিক ও অর্থনৈতিক প্রেক্ষাপটে বেড়ে উঠেছে। আপনার প্রজন্মের কথা চিন্তা করে নিচে প্রদত্ব বাক্যগুলোর সাথে আপনি কতটুকু একমত তা সম্ভাব্য পাঁচটি বিকল্প থেকে নির্বাচিত করুন (টিক চিহ্ন দিন)। মনে রাখবেন, এখানে কোনো ভুল বা সঠিক উত্তর নেই। তাই দয়া করে, অন্যরা কি চিন্তা করছে বা কোনটা সঠিক উত্তর হওয়া উচিত তা না ভেবে নিঃসঙ্কোচে শুধু আপনার নিজস্ব মতামত ব্যক্ত করুন। আপনার সময়ের জন্য আন্তরিক ধন্যবাদ।

১। আমার প্রজন্মের যে অভিজ্ঞতা আছে তা পরবর্তী প্রজন্মের কাছে পৌছানো গুরুত্বপূর্ণ।				
☐<br>একেবারেই একমত না	☐<br>একমত না	☐<br>জানি না	☐<br>একমত	☐<br>সম্পূর্ণ একমত
২। আমি মনে করি আমার প্রজন্ম অনেকের কাছে অর্থপূর্ণ।				
☐<br>একেবারেই একমত না	☐<br>একমত না	☐<br>জানি না	☐<br>একমত	☐<br>সম্পূর্ণ একমত
৩। আমার মনে হয় আমার প্রজন্মকে মানুষ দীর্ঘদিন মনে রাখবে।				
☐<br>একেবারেই একমত না	☐<br>একমত না	☐<br>জানি না	☐<br>একমত	☐<br>সম্পূর্ণ একমত
৪। আমার প্রজন্ম সমাজে অনন্য অবদান রেখেছে।				
☐<br>একেবারেই একমত না	☐<br>একমত না	☐<br>জানি না	☐<br>একমত	☐<br>সম্পূর্ণ একমত
৫। আমার প্রজন্ম তাদের অর্জিত মূল্যবান ধারণা আর অভিজ্ঞতা অন্যদের কাছে ছড়িয়ে দিতে পারে।				
☐<br>একেবারেই একমত না	☐<br>একমত না	☐<br>জানি না	☐<br>একমত	☐<br>সম্পূর্ণ একমত
৬। আমি আমার প্রজন্মের অর্জন নিয়ে গর্বিত।				
☐<br>একেবারেই একমত না	☐<br>একমত না	☐<br>জানি না	☐<br>একমত	☐<br>সম্পূর্ণ একমত
৭। আমি আমার প্রজন্ম নিয়ে ভালো বোধ করি।				
☐<br>একেবারেই একমত না	☐<br>একমত না	☐<br>জানি না	☐<br>একমত	☐<br>সম্পূর্ণ একমত
৮। আমার প্রজন্মের সাথে আমি নিজেকে মেলাতে পারি।				
☐<br>একেবারেই একমত না	☐<br>একমত না	☐<br>জানি না	☐<br>একমত	☐<br>সম্পূর্ণ একমত
৯। আমি নিজেকে আমার প্রজন্মের একজন হিসেবে দেখি।				

☐<br>একেবারেই একমত না	☐<br>একমত না	☐<br>জানি না	☐<br>একমত	☐<br>সম্পূর্ণ একমত
১০। আমি আমার প্রজন্মের একজন গর্বিত সদস্য।				
☐<br>একেবারেই একমত না	☐<br>একমত না	☐<br>জানি না	☐<br>একমত	☐<br>সম্পূর্ণ একমত
১১। আমার প্রজন্ম যেন আমারই এক প্রতিচ্ছবি।				
☐<br>একেবারেই একমত না	☐<br>একমত না	☐<br>জানি না	☐<br>একমত	☐<br>সম্পূর্ণ একমত
১২। আমি আমার প্রজন্মের একজন নিবেদিত সদস্য।				
☐<br>একেবারেই একমত না	☐<br>একমত না	☐<br>জানি না	☐<br>একমত	☐<br>সম্পূর্ণ একমত

Scoring:

- Item 6 to 12 represent Factor 1 (identification with the generation) and item 1 to 5 represent Factor 2 (Awareness of generational importance).
- Assign the following score for each item: 0=Completely disagree, 1=Disagree, 2= Neutral, 3= Agree, 4= Completely agree.
- Sum all the items to get the total GIS score. A higher score indicates a higher generation identity. Similarly, sum the individual factor items to get the total score for each factor.

Citation:

Islam, A., & Haque, S. (2021). Construction and validation of a generational identity scale on Bangladeshi older adults. *Frontiers in Psychology, 12*, Article 703237. https://doi.org/doi: 10.3389/fpsyg.2021.703237

Generational Identity Scale (English version)

Instruction:

Below are some statements about generational identification. By ‘generation’, we refer a group of people who born and raised in a similar historical, social, political and economic condition. Thinking about your generation, please respond to what extent you agree with these statements. There is no right or wrong answer; therefore, try to respond with your best opinion without considering what others are thinking or what might be socially appropriate. We thank you for your time.

1. It is important for my generation to pass along the experiences we have undergone.				
☐ Completely disagree	☐ Disagree	☐ Neutral	☐ Agree	☐ Completely agree
2. I think my generation is meaningful to many people.				
☐ Completely disagree	☐ Disagree	☐ Neutral	☐ Agree	☐ Completely agree
3. I feel that my generation will be remembered for a long time.				
☐ Completely disagree	☐ Disagree	☐ Neutral	☐ Agree	☐ Completely agree
4. My generation has made unique contributions to society.				
☐ Completely disagree	☐ Disagree	☐ Neutral	☐ Agree	☐ Completely agree
5. My generation can pass along valuable ideas and experiences.				
☐ Completely disagree	☐ Disagree	☐ Neutral	☐ Agree	☐ Completely agree
6. I feel proud of my generation’s achievements.				
☐ Completely disagree	☐ Disagree	☐ Neutral	☐ Agree	☐ Completely agree
7. I feel good about my generation.				
☐ Completely disagree	☐ Disagree	☐ Neutral	☐ Agree	☐ Completely agree
8. I identify myself with my generation.				
☐ Completely disagree	☐ Disagree	☐ Neutral	☐ Agree	☐ Completely agree

9. I see me as belonging to my generation.				
☐ Completely disagree	☐ Disagree	☐ Neutral	☐ Agree	☐ Completely agree
10. I am a worthy member of the generation I belong to.				
☐ Completely disagree	☐ Disagree	☐ Neutral	☐ Agree	☐ Completely agree
11. The generation I belong to is an important reflection of who I am.				
☐ Completely disagree	☐ Disagree	☐ Neutral	☐ Agree	☐ Completely agree
12. I am a cooperative participant in the generation I belong to.				
☐ Completely disagree	☐ Disagree	☐ Neutral	☐ Agree	☐ Completely agree

Scoring:

- Item 6 to 12 represent Factor 1 (identification with the generation) and item 1 to 5 represent Factor 2 (Awareness of generational importance).
- Assign the following score for each item: 0=Completely disagree, 1=Disagree, 2= Neutral, 3= Agree, 4= Completely agree.
- Sum all the items to get the total GIS score. A higher score indicates a higher generation identity. Similarly, sum the individual factor items to get the total score for each factor.

Citation:

Islam, A., & Haque, S. (2021). Construction and validation of a generational identity scale on Bangladeshi older adults. *Frontiers in Psychology, 12,* Article 703237. https://doi.org/doi: 10.3389/fpsyg.2021.703237
