# Supplementary figures and images for: Construction and Validation of a Generational Identity Scale on Bangladeshi Older Adults
Source: Front Psychol. 2021 Aug 5;12:703237. doi: 10.3389/fpsyg.2021.703237 (PMC8376147; doi:10.3389/fpsyg.2021.703237)

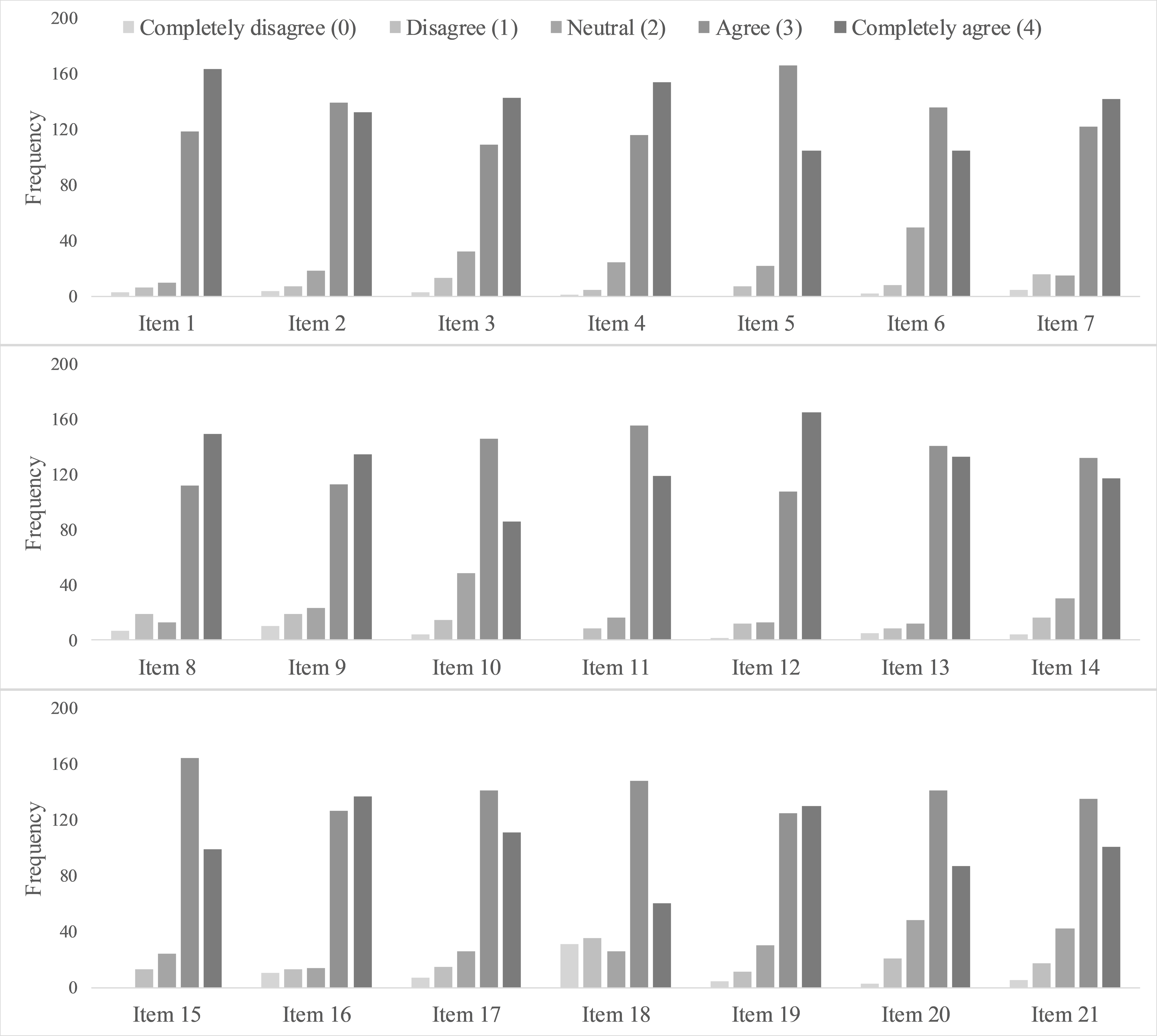

Supplement: Supplementary file 3 [file Image_1.TIF]

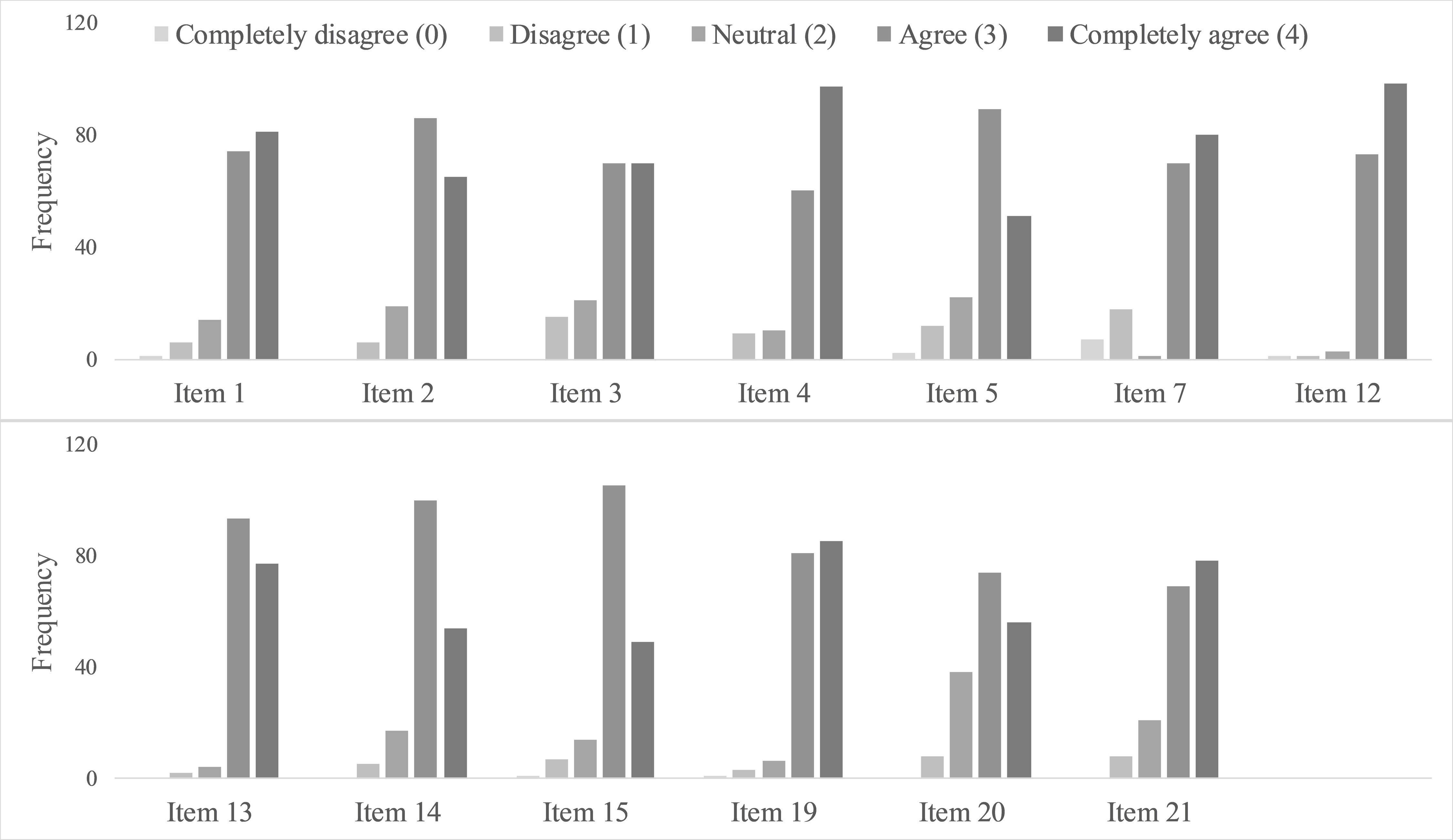

Supplement: Supplementary file 4 [file Image_2.TIF]
